# A Quantitative Model to Estimate Drug Resistance in Pathogens

**DOI:** 10.3390/jof2040030

**Published:** 2016-12-05

**Authors:** Frazier N. Baker, Melanie T. Cushion, Aleksey Porollo

**Affiliations:** 1 Department of Electrical Engineering and Computing Systems, University of Cincinnati, Cincinnati, OH 45221, USA; bakerfn@mail.uc.edu; 2 Center for Autoimmune Genomics and Etiology, Cincinnati Children’s Hospital Medical Center, Cincinnati, OH 45229, USA; 3 Department of Internal Medicine University of Cincinnati College of Medicine, Cincinnati, OH 45267, USA; melanie.cushion@uc.edu; 4 The Veterans Affairs Medical Center, Cincinnati, OH 45220, USA; 5 Department of Biomedical Informatics, University of Cincinnati College of Medicine and Cincinnati Children’s Hospital Medical Center, Cincinnati, OH 45267, USA

**Keywords:** Pneumocystis pneumonia, *Pneumocystis jirovecii*, folate biosynthesis, drug resistance, QSAR, amino acid covariance, amino acid coevolution

## Abstract

Pneumocystis pneumonia (PCP) is an opportunistic infection that occurs in humans and other mammals with debilitated immune systems. These infections are caused by fungi in the genus Pneumocystis, which are not susceptible to standard antifungal agents. Despite decades of research and drug development, the primary treatment and prophylaxis for PCP remains a combination of trimethoprim (TMP) and sulfamethoxazole (SMX) that targets two enzymes in folic acid biosynthesis, dihydrofolate reductase (DHFR) and dihydropteroate synthase (DHPS), respectively. There is growing evidence of emerging resistance by *Pneumocystis jirovecii* (the species that infects humans) to TMP-SMX associated with mutations in the targeted enzymes. In the present study, we report the development of an accurate quantitative model to predict changes in the binding affinity of inhibitors (*K*_i_, *IC*_50_) to the mutated proteins. The model is based on evolutionary information and amino acid covariance analysis. Predicted changes in binding affinity upon mutations highly correlate with the experimentally measured data. While trained on *Pneumocystis jirovecii* DHFR/TMP data, the model shows similar or better performance when evaluated on the resistance data for a different inhibitor of PjDFHR, another drug/target pair (PjDHPS/SMX) and another organism (*Staphylococcus aureus* DHFR/TMP). Therefore, we anticipate that the developed prediction model will be useful in the evaluation of possible resistance of the newly sequenced variants of the pathogen and can be extended to other drug targets and organisms.

## 1. Introduction

Pneumocystis pneumonia (PCP) is a potentially lethal fungal infection affecting patients with an incompetent immune system, including patients with AIDS, autoimmune disorders, and after organ transplantation, as well as other morbidities requiring medically induced suppression of the immune system. The cause of PCP in humans is *Pneumocystis jirovecii*, which like other fungi in the genus *Pneumocystis*, do not respond to the commonly used anti-fungal therapies. A combination therapy, consisting of trimethoprim and sulfamethoxazole (TMP-SMX), which targets the folate biosynthesis pathway at 2 enzymatic steps, remains the primary option for the treatment and prophylaxis of PCP.

In addition to concerns about the toxicity of the TMP-SMX treatment [1] and low tolerance to sulfa-based drugs in some patients [2], there is growing evidence of the emerging resistance of the fungi associated with the acquired mutations in the targeted enzymes. Earlier studies simply reported sequence variants found in *P. jirovecii* DHPS and DHFR suggesting the development of resistance upon exposure to the drug [3,4]. Later, as the number of PCP patients unresponsive to TMP-SMX increased and the corresponding strains of the pathogen were sequenced, it became possible to draw statistically significant associations and estimate possible risks of resistance upon prior exposure to the drug [5,6,7,8,9,10,11]. Finally, in vitro enzymatic assays and PjDHPS/PjDHFR heterologous systems based on the respective knockouts in *Saccharomyces cerevisiae* enabled the measurement of the kinetic parameters of these enzymes with the wild type sequence and identified mutations [12,13,14,15,16,17].

Recently, a new quantitative model has been suggested to estimate the effect of missense mutations on drug resistance [18]. The model is based on a massive experiment with *Escherichia coli* treated with amoxicillin, followed by the sequencing of mutations in beta-lactamase (TEM-1) and measurement of the corresponding enzymatic activity [19]. It has been shown that the model used to predict drug resistance based on a combination of individual position specific amino acid probabilities with the amino acid co-variance scores outperforms SIFT [20], PolyPhen2 [21], and a set of methods predicting the effect based on the estimated change in stability of the mutated proteins (I-Mutant [22], MUpro [23], and PoPMuSiC [24]) [18]. Co-variance scores reveal pairwise concerted changes of amino acids at different positions within a protein sequence and may represent “epistatic” interactions between the residues. Both position specific probabilities and co-variance scores are derived from the multiple sequence alignments (MSA). In this published model, co-variance scores are computed using one of the most advanced methods in the field of protein co-evolution analysis, Direct Coupling Analysis (DCA), which employs approaches from statistical thermodynamics to delineate direct and transient co-variance relationships between residues at different positions in the protein [25]. However, the complexity of the DCA method brings certain limitations to applicability of the presented quantitative drug resistance model. It requires extensive multiple sequence alignments, deals with well-defined domains only, cannot process multi-domain proteins and sequences longer than 500 amino acids, and is very computationally intensive [18,25,26]. For example, when evaluating the DCA-based model on TEM-1 data, only a fraction of mutations were considered, specifically, those that fell in the Pfam domain and represented single mutations [18].

We have recently developed a new tool for the amino acid co-variance analysis, CoeViz [27] that overcomes most of the limitations listed above for DCA. In particular, CoeViz is not limited by the protein domains nor the large size of the MSA, can handle proteins of any length in a practical time frame, and generates co-variance scores using three metrics: Mutual Information (MI), Chi-squared (*χ^2^*), and Pearson correlation (*r*). The tool accounts for phylogenetic bias in the MSA and also provides an alternative way of adjusting the scores for MI using the average product correction (APC [28]).

In this work, we have built a new model to evaluate the effect of mutations on resistance to drugs. In contrast to the DCA-based model, our approach considers the entire protein sequence and estimates the relative effect of mutations compared to the reference sequence. Moreover, our model can compute the effect of multi-position variants by considering them simultaneously. The new model was trained on the kinetics data of the PjDHFR inhibition by TMP and was further evaluated using experimental data for a different inhibitor targeting PjDHFR (OAAG324 [17]) as well as inhibition data for PjDHPS, *Staphylococcus aureus* DHFR, to estimate generalization of the model to different drugs, drug targets, and organisms.

## 2. Materials and Methods

### 2.1. Formulation of the Quantitative Model

It has recently been suggested to use the difference in protein “phenotype” (fitness function) between the mutant and a wild type sequence as the estimate of drug resistance elicited by mutations (Equation (1), [18]).

(1) $$\Delta \Phi \left(a_{i}\to b\right)=\Phi \left(a_{1},\ldots ,a_{i-1},b,a_{i+1},\ldots ,a_{L}\right)-\Phi \left(a_{1},\ldots ,a_{i-1},a_{i},a_{i+1},\ldots ,a_{L}\right),$$

where $\Delta \Phi \left(a_{i}\to b\right)$ is a change in protein function (drug resistance or enzymatic activity in the context of this work) upon mutation of amino acid *a* to *b* at position *i* in the protein sequence of length *L*. Protein function Φ can be further defined by the two groups of parameters, derived from the individual positions (Φ*_IND_*) and pairwise “epistatic interactions” of amino acids (Φ*_EPI_*), Equations (2) and (3).

(2) $$\Phi_{IND}\left(a_{1},\ldots ,a_{L}\right)=\overset{L}{\underset{i=1}{\sum }}\varphi_{i}\left(a_{i}\right)$$

(3) $$\Phi_{EPI}\left(a_{1},\ldots ,a_{L}\right)=\underset{1\leq i\leq j\leq L}{\sum }\varphi_{ij}\left(a_{i},a_{j}\right)$$

where *ϕ_i_*(*a_i_*) is a single site term and *ϕ_ij_*(*a_i_*,*a_j_*) defines “epistatic coupling” between positions *i* and *j*. They are derived from joint pairwise probabilities observed in the MSA, refer to [18] for further details in their definition. Then, the change in protein function upon mutation at position *i* can be defined as

(4) $$\Delta \Phi_{DCA}\left(a_{i}\to b|a_{1},\ldots ,a_{i-1},a_{i+1},\ldots ,a_{L}\right)=\varphi_{i}\left(b\right)-\varphi_{i}\left(a_{i}\right)+\overset{L}{\underset{j=1}{\sum }}\left[\varphi_{ij}\left(b,a_{j}\right)-\varphi_{ij}\left(a_{i},a_{j}\right)\right]$$

Equation (4) represents the model proposed by Figliuzzi and colleagues [18] to quantify the mutation effect on a protein function. While it was estimated to outperform other existing methods for the prediction of mutation effect in the given enzyme (TEM-1), there are at least two shortcomings of this approach. First, the model considers single mutations only. It has not been applied to predict the effect of multiple missense mutations in proteins. This seems to be counter-intuitive given that the model has access to all pairwise coupling scores, so it could be extended to the estimates of compensatory or stabilizing simultaneous mutations. Second, it considers both individual positions and all pairwise epistatic interactions contributing equally to the scoring function. However, some sites and residue couplings may be more important for protein function or structural stability than the others.

We have developed a new model that considers all mutations found in a given strain simultaneously and incorporates weights for each pair of comparisons. The corresponding weighted models ($\Delta \Phi_{WIND}$ and $\Delta \Phi_{WEPI}$) to quantify the change in protein “phenotype” are defined as follows:

(5) $$\Delta \Phi_{WIND}\left(A\to B\right)=\underset{i\in M}{\sum }w_{i}\varphi_{i}\left(b_{i}\right)$$

(6) $$\begin{array}{c} \Delta \Phi_{WEPI}\left(A\to B|WT\backslash A\right)=\\ \;\;\;\;\left(\underset{i\in M}{\sum }\underset{\begin{array}{c} 1\leq j\leq L\\ j\notin M \end{array}}{\sum }w_{ij}\varphi_{ij}\left(b_{i},a_{j}\right)-\underset{i\in M}{\sum }\underset{\begin{array}{c} 1\leq j\leq L\\ j\notin M \end{array}}{\sum }w_{ij}\varphi_{ij}\left(a_{i},a_{j}\right)\right)+\\ \left(\underset{\begin{array}{c} i,j\in M\\ i<j \end{array}}{\sum }w_{ij}\varphi_{ij}\left(b_{i},b_{j}\right)-\underset{\begin{array}{c} i,j\in M\\ i<j \end{array}}{\sum }w_{ij}\varphi_{ij}\left(a_{i},a_{j}\right)\right) \end{array}$$

where *A* is a set of amino acids at positions *M* that underwent mutations *B*; *WT* are amino acids in the reference (wild type) sequence. $\varphi_{i}\left(a_{i}\right)$ is a similarity score for the amino acid *a_i_*, which is taken from a dedicated position specific similarity matrix (PSSM), generated by PSI-BLAST [29], and represents the likelihood of finding a given amino acid substitution at a given position); $\varphi_{ij}\left(a_{i},a_{j}\right)$ is a joint probability of the amino acids at positions *i* and *j* derived from the MSA. *w_i_* is a weight for an individual position *i* computed as 1—normalized Shannon entropy (*S*, Equation (7)), and *w_ij_* is a weight for epistatic interaction between the residues at positions *i* and *j* computed using one of the co-variance metrics (MI, χ^2^, or *r*; see full definitions and final score transformations in [27]), all adjusted for phylogeny bias in the MSA.

(7) $$w_{i}=1-S_{i}=1-\frac{1}{log\left(20\right)}\overset{20}{\underset{j=1}{\sum }}p_{ij}\log p_{ij}$$

where *p_ij_* is the probability of the amino acid type *j* to occur at position *i*, derived from the MSA. MSAs and associated PSSMs are generated using PSI-BLAST with three iterations against the NCBI nr database. PSI-BLAST is parameterized to report 2000 sequences in the final MSA, but the model can work with the fewer number of sequences.

To have “phenotype” scores (∆Φ) always positive and in the range [0,1], both models are normalized using the logistic sigmoid function defined in Equation (8).

(8) $$\Delta \Phi_{N}=1-\frac{1}{1+e^{-\frac{\Delta \Phi }{10}}}$$

The final model ($\Delta \Phi_{WDR}$) to estimate the change in drug resistance upon mutations in the targeted protein is the product of normalized individual- and pairwise epistatic interactions-based models (Equation (9)).

(9) $$\Delta \Phi_{WDR}=\Delta \Phi_{NWIND}\times \Delta \Phi_{NWEPI}$$

### 2.2. Experimental Data

The quantitative model was trained and evaluated using kinetic data (*K*_i_, *IC*_50_) for the following drug targets and their mutants (Table 1).

Since the values of the kinetic parameters vary between mutants by 2–3 orders of magnitude, all experimental data are log_10_ transformed. It is also more informative to operate with relative values when comparing mutants to reference sequences. Hence, we converted the data to the relative change in kinetics of inhibition (Equation (10)).

(10) $$\Delta K=\frac{\log_{10}K\left(Mut\right)-\log_{10}K\left(Ref\right)}{\left|\log_{10}K\left(Ref\right)\right|}$$

where *K* is an experimentally measured kinetic parameter of inhibition (*K*_i_ or *IC*_50_) for the reference (Ref) and mutated (Mut) protein.

Performance of the models is evaluated using the Pearson correlation coefficient. Given the limited number of data points, all results are based on the jackknife resampling and reported in the mean ± SD format.

## 3. Results

### 3.1. Choice of the Model

To choose a quantitative model that demonstrates the strongest correlation to experimental data, we used kinetic data for the inhibition of PjDHFR variants with TMP [17]. This is the largest dataset available for drug targets and their variants in *P. jirovecii*. The dataset contains 13 single variants and 6 double mutants of PjDHFR. Different models described in Methods were evaluated varying the co-variance metrics for the $\Phi_{EPI}$ component. Table 2 provides a summary of the evaluated models.

**Figure 1:**
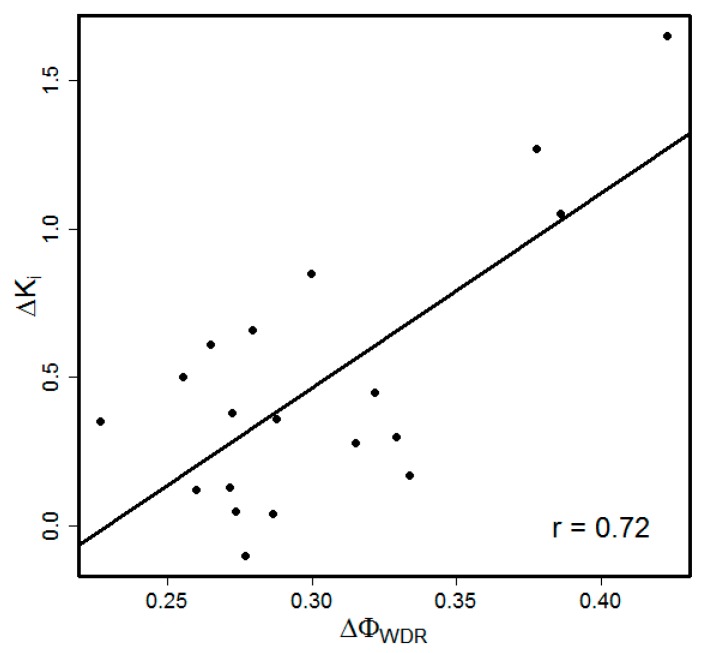
Correlation between the scores produced by the $\Delta \Phi_{WDR}$ model and experimental kinetic data for inhibition of PjDHFR variants by TMP. *K*_i_ values are log_10_ transformed and converted to a change relative to inhibition kinetics of the reference PjDHFR.

The top performing model yielding *r* = 0.72 appears to be $\Delta \Phi_{WDR}$ that incorporates mutual information-based metric adjusted with the average product correction (APC). Figure 1 illustrates correlation between the experimental data and this model.

### 3.2. Evaluation of the Model

We tested the generalization of the model using different inhibitors, mutations that were not the result of drug treatment, the same drug target in a distant organism (fungal vs bacterial), another drug/drug target pair in the same organism (*P. jirovecii*). Table 3 summarizes the results of the proposed quantitative model.

Queener and colleagues tested whether naturally occurring variants in PjDHFR, resistant to TMP inhibition, would show similar resistance to other PjDHFR inhibitors by choosing another competitive inhibitor, OAAG324, to evaluate [17]. Using the structure modeling of PjDHFR, they determined that OAAG324 has a different binding mode to the enzyme than TMP, involving a different set of amino acids at the active site. Experimental data indicated that the majority of the PjDHFR variants remained sensitive to OAAG324 suggesting that mutations in the enzyme are naturally selected to compensate for the pressure from a given inhibitor and may not necessarily be beneficial against other inhibitors with a different binding mode. This is also reflected by our model, whose performance drops from *r* = 0.72 to 0.60 for the same set of mutations, when switching from TMP to OAAG324.

The same group of authors went further and modified selected positions at the active site of PjDHFR to make it more similar to the DHFR of *P. carinii* (a species that infects rats) or to that of the human enzyme. Kinetic constants of inhibition were measured for the same two compounds, TMP and OAAG324 [16]. These mutations do not represent natural selection under pressure of TMP, therefore no high correlation between the model and experimental data was anticipated. Nevertheless, our model showed moderate correlation for TMP (*r* = 0.41) and anti-correlation for OAAG324 (*r* = −0.39).

Of greater interest was whether the model could be applied to evaluate mutations that emerged under the pressure of the same drug within a different organism and to a different pair of drug/drug target. In both cases, our model showed strong correlation with the experimental data. Its prediction of the change in resistance upon DHFR mutations in *S. aureus* after the TMP treatment [30] yielded *r* = 0.91, whereas the predicted effect of mutations on resistance of PjDHPS after the SMX treatment [13] correlated well with the experimental data (*r* = 0.79).

We could not compare the performance of our model with the DCA-based model due to multiple obstacles: the published model has no implementation to use it online or as a stand-alone software; it considers conserved domains only and relatively short sequences, while PjDHPS (UniProt ID: L0P7Z1) is 742 amino acids long; it is not applicable to multi-position variants. We also could not evaluate PolyPhen2 as the web-server was returning an error that all the specified UniProt IDs are not part of the available version of UniProtKB. We did not evaluate the protein stability-based methods for the prediction of mutation effects as (1) they were already shown to be not applicable to the drug resistance data [18]; (2) the mutated enzymes are fully functional given that they are essential and the sequenced resistant strains were viable. Therefore, we compared our model with SIFT. For variants with multiple simultaneous mutations, a sum of individual SIFT scores was computed. As can be seen from Table 3, SIFT is not appropriate for evaluation of resistance in drug targets conferred through mutations.

## 4. Discussion

Pathogens facing selective pressure, such a drug therapy or prophylaxis treatment, are able to develop resistance to the drug through the concerted mutations impeding the binding of an inhibitor or alleviating its action while retaining the essential function of the targeted endogenous protein. PCP exemplifies the problem of emerging resistance when the repertoire of therapeutics is limited. With the advent of the targeted sequencing, it is now possible to quickly identify mutations in the resistant strain of the pathogen. However, the comparative evaluation of these variants on the drug susceptibility is lagging. We have developed a quantitative model that accounts for both individual changes and concerted mutations in the drug target to predict a protein’s resistance to an inhibitor.

Drug resistance, in general, and resistance to antifolates, in particular, may be conferred through alternative mechanisms. In addition to compensatory mutations [12,31], pathogens may employ a drug-targeted gene amplification [32,33], reduction of cell wall permeability to the drug or encoding alternative forms of the targeted gene [34], or activation of the ATP-binding cassette (ABC) transporters and multidrug resistance genes (MDR) to efflux drugs out of cell [35,36,37]. Obviously, the proposed model cannot account for these strategies of resistance. Therefore, it most likely will not strongly correlate with the minimal inhibitory concentrations (MIC) commonly used to evaluate the overall drug resistance by a given pathogenic strain. MIC may be a complex function of the drug compensatory mechanisms mentioned above, where mutations in the targeted protein may be important but are not a major factor determining the overall resistance.

Other limitations of the proposed model include inability to quantify variants with insertions and deletions, as well as other mutations unrelated to drug resistance; lack of strong correlation of predictions to the kinetic data for inhibitors possessing a mode of action different than the one(s) a drug target to which it has developed its resistance. Nevertheless, the model may help evaluate and compare resistant strains with known variants in the targeted protein and facilitate predictions of possible resistance conferred through concerted compensatory missense mutations. Such an approach would be quite valuable in microbial systems like *Pneumocystis*, which do not have an in vitro cultivation system that could be used for such predictions. 

## 5. Conclusions

The presented quantitative model shows accurate performance in predicting the change in resistance of the mutated proteins under the pressure of a drug treatment. In contrast to the existing methods for evaluating the mutation effect, our model does not predict the damaging effect of the mutation but rather estimates the fitness of the mutated protein to withstand exposure to an inhibitor while maintaining a vital function in the organism. Contrary to other methods that consider one mutation at a time, our model can evaluate multiple simultaneous mutations, which may represent concerted compensatory changes in the protein. The model appears to be transferrable among distant organisms and different drug targets. Tools such as this model contribute to our armamentarium against the evolving defenses of microbial pathogens and should help in the earlier detection of these evasive strategies. We envision at least two directions where the presented prediction model can be utilized. (1) To quantitatively inform researchers and clinicians about the resistance level of sequenced strains conferred through mutations to a given drug. (2) To predict possible mutations (and their rate) a pathogen may acquire to compensate for the inhibitory effect of a drug.

## Figures and Tables

**Table 1:** Experimental data used to build and evaluate the model for the estimation of drug resistance.

Organism Protein (UniProt ID)	Mutants	Kinetic Parameter	Refs.	Purpose (Drug/Inhibitor)
*P. jirovecii* DHFR (Q9UUP5)	T14A, P26Q	*K*_i_	[17]	Train the model on one target (TMP)
	N23S			
	S31F			
	F36C			
	L65P			
	F36C, L65P			
	S37T			
	A67V			
	A67V, C166Y			
	R59G, A67V			
	V79I			
	S106P			
	S106P, E127G			
	T144A			
	T144A, K171E			
	D153V			
	I158V			
	C166Y			
	R170G			
*P. jirovecii* DHFR (Q9UUP5)	T14A, P26Q	*K*_i_	[17]	Evaluate on the same target, different inhibitor (OAAG324)
	N23S			
	S31F			
	F36C			
	L65P			
	F36C, L65P			
	S37T			
	A67V			
	A67V, C166Y			
	R59G, A67V			
	V79I			
	S106P			
	S106P, E127G			
	T144A			
	T144A, K171E			
	D153V			
	I158V			
	R170G			
*P. jirovecii* DHFR (Q9UUP5)	S69F	*K*_i_	[16]	Evaluate on the same target, artificially mutated (TMP, OAAG324)
	S37K, S69F			
	S37Q			
	S69N			
	S37Q, S69N			
	S37K, S69N			
	S37Q, S69F			
*S. aureus* DHFR (P0A017)	F99Y	*IC*_50_	[30]	Evaluate on the same target, different organism (TMP)
	H31N, F99Y			
	F99Y, H150R			
	L21V, N60I, F99Y			
*P. jirovecii* DHPS (L0P7Z1)	T519A	*IC*_50_	[13]	Evaluate on a different drug target, different drug (SMX)
	P521S			
	T519A, P521S			
	T519V, P521S			

**Table 2:** Performance of the quantitative models in terms of Pearson correlation with experimental data for PjDHFR (19 variants) inhibited by trimethoprim (TMP). *

Co-Variance Metric	$\Delta \Phi_{IND}$	$\Delta \Phi_{EPI}$	$\Delta \Phi_{WIND}$	$\Delta \Phi_{WEPI}$	$\Delta \Phi_{NWIND}$	$\Delta \Phi_{NWEPI}$	$\Delta \Phi_{WDR}$
*χ^2^*	−0.67 ± 0.05	−0.53 ± 0.05	−0.62 ± 0.05	−0.29 ± 0.05	0.68 ± 0.04	0.29 ± 0.05	0.64 ± 0.04
MI	−0.67 ± 0.05	−0.53 ± 0.05	−0.62 ± 0.05	−0.05 ± 0.05	0.68 ± 0.04	0.05 ± 0.05	0.70 ± 0.04
APC (MI)	−0.67 ± 0.05	−0.53 ± 0.05	−0.62 ± 0.05	−0.28 ± 0.06	0.68 ± 0.04	0.28 ± 0.06	0.72 ± 0.05
*r*	−0.67 ± 0.05	−0.53 ± 0.05	−0.62 ± 0.05	−0.20 ± 0.07	0.68 ± 0.04	0.20 ± 0.07	0.65 ± 0.05

* Models independent of co-variance metrics have constant results and included here for comparison.

**Table 3:** Performance of the models on diverse datasets.

Target	Inhibitor	The Number of Mutations: Single/Double/Triple	$\Delta \Phi_{WDR}$	SIFT
PjDHFR	OAAG324	12/6/0	0.60 ± 0.03	−0.22
^1^ PjDHFR->PcDHFR/HsDHFR	TMP	3/4/0	0.41 ± 0.10	−0.48
^1^ PjDHFR->PcDHFR/HsDHFR	OAAG324	3/4/0	−0.39 ± 0.16	−0.07
SaDHFR	TMP	1/2/1	0.91 ± 0.12	−0.59
PjDHPS	SMX	2/2/0	0.79 ± 0.18	ND ^2^

^1^ Mutations artificially introduced in PjDHFR to make its active site similar to either *P. carinii* or human DHFR. ^2^ SIFT predicted all mutations to be damaging with score = 0; *r* cannot be computed (SD = 0).
